# Ballistic Thermal Transport in Carbyne and Cumulene with Micron-Scale Spectral Acoustic Phonon Mean Free Path

**DOI:** 10.1038/srep18122

**Published:** 2015-12-10

**Authors:** Mingchao Wang, Shangchao Lin

**Affiliations:** 1 Department of Mechanical Engineering, Materials Science and Engineering Program, FAMU-FSU College of Engineering, Florida State University, Tallahassee, Florida 32310, USA

## Abstract

The elastic modulus of carbyne, a one-dimensional carbon chain, was recently predicted to be much higher than graphene. Inspired by this discovery and the fundamental correlation between elastic modulus and thermal conductivity, we investigate the intrinsic thermal transport in two carbon allotropes: carbyne and cumulene. Using molecular dynamics simulations, we discover that thermal conductivities of carbyne and cumulene at the quantum-corrected room temperature can exceed 54 and 148 kW/m/K, respectively, much higher than that for graphene. Such conductivity is attributed to high phonon energies and group velocities, as well as reduced scattering from non-overlapped acoustic and optical phonon modes. The prolonged spectral acoustic phonon lifetime of 30–110 ps and mean free path of 0.5–2.5 μm exceed those for graphene, and allow ballistic phonon transport along micron-length carbon chains. Tensile extensions can enhance the thermal conductivity of carbyne due to the increased phonon density of states in the acoustic modes and the increased phonon lifetime from phonon bandgap opening. These findings provide fundamental insights into phonon transport and band structure engineering through tensile deformation in low-dimensional materials, and will inspire studies on carbyne, cumulene, and boron nitride chains for their practical deployments in nano-devices.

Advanced thermal management and heat dissipation using thermally conductive nanomaterials have become an emergent field which attracts many attentions in the past decade^1^,^2^. Recent efforts in searching material candidates have led to the discovery, understanding and deployment of many highly conductive materials, ranging from 2D graphene^3^ and hexagonal boron nitride (h-BN)^4^ to 1D carbon nanotubes^5^, single polymer chains^6^,^7^ and polymer nanofibers^8^,^9^. For example, experimentally reported thermal conductivity can reach ~5 kW/m/K for suspended monolayer graphene^3^, and ~3.5 kW/m/K for suspended metallic single-walled carbon nanotubes (SWCNTs)^5^. Such high thermal conductivity is closely related to their oustanding mechanical properties, in particular, the ultrahigh elastic modulus of ~1 TPa for both graphene^10^ and CNTs^11^. This correlation could be explained from classical theories such as the Cauchy-Born rule, in which the elastic modulus *E* is directly proportional to the interatomic chemical bonding stiffness *K*. High bonding stiffness can lead to materials with high phonon energy *ħω* (the frequency *ω* ~ *K*^*1/2*^ for wave vectors *k* → 0) and group velocity (*v* ~ *E*^*1/2*^ for *k* → 0), which ultimately contribute to their high thermal conductivities *κ* based on the kinetic theory for phonon transport^12^. From the aspect of intrinsic spectral phonon properties, low-dimensional carbon-based materials possess simple vibrational modes and phonon band structures, which should greatly reduce the magnitude of phonon-phonon scattering. However, even 2D graphene exhibits highly overlapped acoustic and optical phonon branches^13^, which generates additional phonon scattering events. Theoretically, 1D linear atomic chains should possess perfectly non-overlapped phonon branches^12^ and therefore, exhibit greatly enhanced thermal conductivity compared to graphene, but such material has not been observed yet using computations or experiments.

Very recently, ultrahigh elastic modulus was reported, using molecular dynamics (MD) simulations^14^ and first-principle calculations^15^, for carbyne, an *sp*-hybridized carbon allotrope which can be viewed as a 1D linear acetylenic carbon chain (Fig. 1(a)). Its Young’s modulus *E* was reported to be in the range of 3 ~ 32.7 TPa, about one order of magnitude higher than graphene and SWCNTs. Its superlative elastic modulus motivates us to explore its potential ultrahigh thermal conductivity, as one would expect it to be much higher than graphene and SWCNTs. Furthermore, in addition to recent reports on free-standing single carbon^16^ and BN chains^17^ etched from graphene and h-BN using electron beams, respectively, tremendous efforts have been made on chemical syntheses, growths and characterizations of stable and long 1D atomic chains^18^,^19^. Interestingly, it has been discovered that multi-walled carbon nanotubes (MWCNTs) can serve as reaction capsules to synthesize stable linear carbon chains (up to 100 carbon atoms) embedded in their hollow cylindrical cores^20^,^21^. Not only are these atomic chains of fundamental interests in nanoscale phonon transport, but they also have many potential applications in ultra-compact nano-electronic/spintronic devices^22^,^23^. Considering the instability of pure carbon chains in the condensed phase (exothermal cross-linking)^24^, encapsulating single carbon chains in MWCNTs could lead to very stable and highly efficient thermal dissipation junctions, metal-metal/metal-semiconducting junctions, and diodes in nano-electronics^20^.

In this work, we investigate phonon thermal transport in a single carbyne chain (polyyne, (−C≡C−)_n_) using equilibrium MD simulations. Another form of the 1D carbon chains, cumulene ((=C = C=)_n_, Fig. 1(b))^25^, is also studied here for comparison, knowing that it is less stable than carbyne and will undergo a Peierls transition into carbyne^26^. The effects of simulation cell size (chain length), temperature (quantum-corrected) and tensile strain are investigated. Intrinsic spectral (mode-specific) phonon properties, such as phonon density of states (DOS), dispersion relation, group velocity, lifetime, and mean free path (MFP), are also predicted and compared with analytical lattice dynamics (LD) calculation results to better understand the predicted ultrahigh thermal conductivities. Our analyses confirm that such ballistic thermal transport is attributed to high phonon frequencies, group velocities and phonon lifetime. The resulting phonon MFP is primarily in the range of 0.5 to 2.5 m for both carbyne and cumulene. Based on the kinetic theory of phonon transport, we confirmed that the major contribution to the total conductivity comes from the longitudinal acoustic mode. Besides, tensile extensions can enhance the thermal conductivity of carbyne, partially due to the enhanced DOS in low-frequency acoustic modes as well as the increased phonon lifetime.

## Results and Discussion

### Temperature-Dependent Thermal Conductivity

Considering the fact that chemically synthesized free-standing carbyne chains are still short (up to 44 atoms^18^) with end capping groups, we study relatively short chains from 24 (~3.3 nm) to 192 carbon atoms (~26.2 nm). The convergence of thermal conductivity and heat flux autocorrelation function (HFACF) are confirmed at various chain lengths (see Methods Section and Supplementary Fig. S1). We find that the predicted values of *κ* show weak dependence on simulation cell size because the Green-Kubo approach (see Methods Section) only rules out low *k* phonon modes that have small contributions to the total *κ* value^27^. Therefore, all data analyses hereafter are based on carbyne and cumulene chains of around 13.1 nm (96 carbon atoms). The predicted *κ* is equal to 80 ± 26 kW/m/K for carbyne and 200 ± 52 kW/m/K for cumulene at 200 ~ 300 K (Fig. 2), more than one order of magnitude higher than suspended monolayer graphene (~5 kW/m/K)^3^ and SWCNTs (~3.5 kW/m/K)^5^. It is also noteworthy that both carbon chains have finite thermal conductivities determined by the Green-Kubo method. They are different from the free 1D Fermi-Pasta-Ulam (FPU) chains^28^,^29^ which exhibit diverging displacements orthogonal to the longitudinal direction. This is because that the FPU chains do not possess angular bending (three-body) potentials to constrain the large-amplitude fluctuations of the chain, while the force fields used here for carbyne and cumulene are more accurate and realistic by considering the bending stiffness of both chains (see Methods Section).

The Debye temperature *T*_D_ for carbyne and cumulene is: *T*_D_ ≈ ℏ*ω*_D_/*k*_B_, where *ω*_D_ = 370 THz is the Debye frequency (maximum excitable phonon frequency obtained from phonon DOS or dispersion), and ℏ is Planck’s constant. The resulting *T*_D_ ≈ 2800 K for both carbyne and cumulene, which is higher than that for graphene and other carbon allotropes^30^ and consistent with Raman spectroscopic results^31^ and first-principle calculations^32^,^33^. Therefore, quantum corrections (QC)^34^ are required to convert the MD temperature, *T*_MD_, into the realistic QC temperature, *T*_QC_, following previous reports^35^. Specifically,


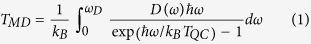


where *D*(*ω*) is the normalized total phonon DOS determined from MD simulations (at *T*_MD_ = 300 K and we ignore the variations in linewidths at different temperatures, and see Methods Section); [exp(ℏ*ω*/*k*_B_*T*_*QC*_)–1]^−1^ is the Bose-Einstein distribution for phonon excitation; *ω* is angular frequency, and *k*_B_ is the Boltzmann constant. The temperature conversion curves are shown in Supplementary Fig. S2, suggesting that the QC room temperature, *T*_QC_ = 300 K, corresponds to an MD temperature of *T*_MD_ = 43 K for carbyne and 48 K for cumulene. Thermal conductivity can also be predicted using the kinetic theory for phonon transport as a function of *ω* or in the reciprocal space (k-space). Specifically, in the k-space with polarizations,


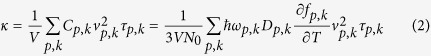


where *C*_*p*,*k*_ is the phonon specific heat, *τ*_*p*,*k*_ is the phonon lifetime, *V* is the system volume, *N*_0_ is the total number of atoms in the system, *v*_*p,k*_ is the phonon group velocity, *D*_*p,k*_ is the phonon DOS, and *f*_*p*,*k*_ is the Bose-Einstein distribution for phonon excitations. Subscripts ‘*p*’ and ‘*k*’ denote the phonon polarization and wave vector, respectively.

MD predicted *κ* of carbyne and cumulene at different *T*_MD_ and *T*_QC_ are shown in Fig. 2 in log scales. The figure indicates that there exists strong quantum effects at low MD temperatures (0 ~ 100 K) and that the quantum effect gets weaker with increasing temperature. Although quantum corrections are not perfect, the primary conclusions presented in this work and the observed trends of thermal conductivity as a function of temperature should still hold. The observed positive temperature dependence of *κ* at low temperatures agrees very well with the kinetic theory of phonon transport^36^, in which the contribution from *C*_*p*,*k*_ (~ *T* 3 at low temperatures) to *κ* is dominant. The observed negative temperature dependence of *κ* at higher temperatures is also consistent with the increased phonon-phonon scattering when more phonon modes are excited. The excitingly high *κ* value for carbyne is two orders of magnitude higher than single-chain polyethylene (>0.1 kW/m/K)^6^, and one order of magnitude higher than monolayer graphene at the room temperature (~ 5 kW/m/K)^3^. At very low temperatures (*T*_QC_ = 100 K) where cumulene could be chemically stable, it still holds a high *κ* value of ~ 5 kW/m/K, comparable to that of monolayer graphene at the room temperature. At finite temperatures, there exist small displacement fluctuations in both carbon chains, which can break the symmetry of the linear 1D structure. Tensile extensions of the chain can further suppress such fluctuations, while tensile compressions can lead to enhanced, intermediate fluctuations. Nevertheless, our Green-Kubo analysis confirms that the heat flux and the corresponding thermal conductivity along the *x* and *y*-axes are much lower than (<1% of) those along the *z*-axis (longitudinal direction), and therefore, can be neglected. So here we only focus on the *k*-vectors along the *z*-axis and calculate the corresponding phonon dispersions, which is the primary contribution to the total thermal conductivity.

### Intrinsic Spectral Phonon Transport Properties

**Figure 1:**
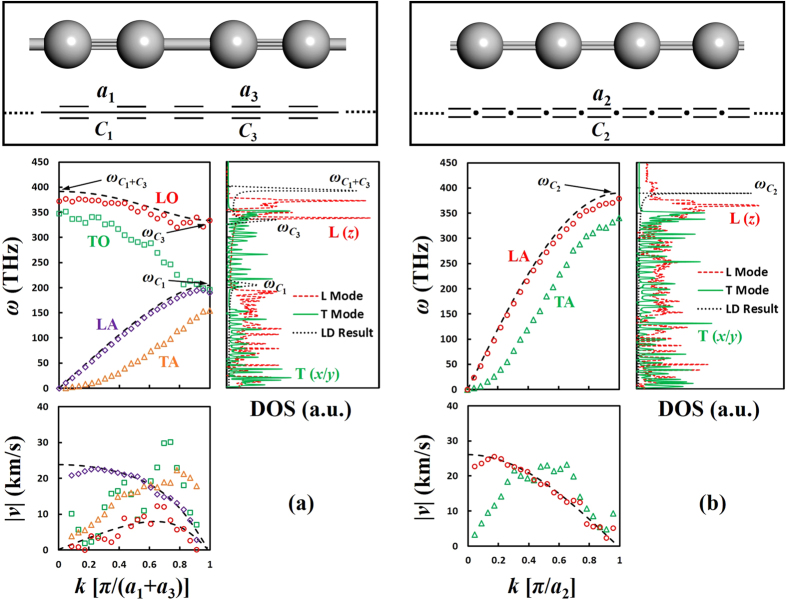
Atomic and chemical structures (top), phonon dispersion (middle left), phonon DOS (middle right) and absolute group velocity (bottom left) of a single (a) carbyne chain and (b) cumulene chain. MD results are shown in colored symbols (for dispersion and velocity) or lines (for DOS), while LD results are shown in black dashed (for dispersion and velocity) or dotted lines (for DOS). The same color and symbol code is applied to each polarization. Characteristic phonon modes predicted by LD are marked in the dispersion and the DOS plots.

**Figure 2:**
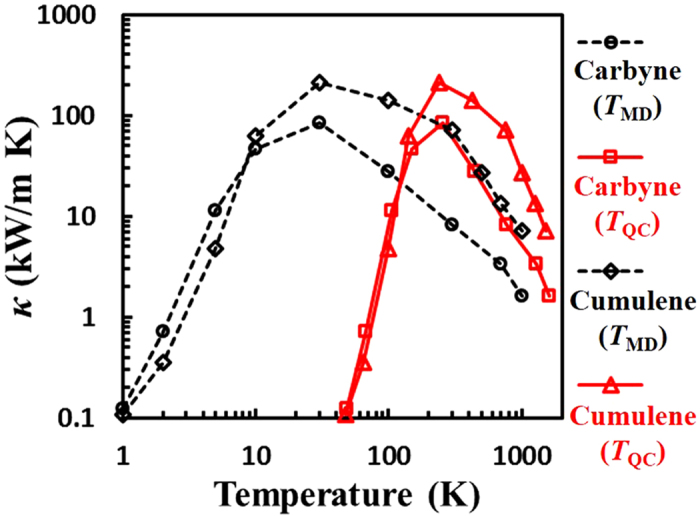
MD predicted thermal conductivity *κ* as a function of temperature (*T*_MD_ and *T*_QC_) for carbyne and cumulene. Standard errors (not shown) in *κ* are in the range of 25% ~ 40% based on three independent simulations.

To elucidate the underlying mechanism of thermal transport in carbyne and cumulene, we carefully investigate their phonon properties derived from MD simulations at 300 K (See Methods Section). Simulated phonon DOS (Fig. 1) unveils the range of possible angular frequencies for both carbon chains: *ω* = 0 ~ 400 THz. The upper limit (400 THz) is close to that from measured Raman spectra (2100 cm^−1^ ≈ 394 THz), corresponding to the high-frequency carbon-carbon bond stretching mode^37^. The phonon dispersion of carbyne (Fig. 1(a)) reflects a monoatomic chain with two spring constants, similar to that of a diatomic chain with two atomic masses^36^. The L modes at *k* = 0 and at the boundary of the first Brillouin zone (also reflected from the three peaks in the longitudinal phonon DOS) correspond to the effective spring constants of C−C bond (*C*_1_/2), C≡C bond (*C*_3_/2), and their combination ((*C*_1_ + *C*_3_)/2) (see Fig. 1(a) and Supplementary Information). The T modes at *k* = 0 and at the boundary of the first Brillouin zone also match well the transverse phonon DOS. The longitudinal optical (LO) mode in carbyne (330 ~ 370 THz, due to the C≡C and the combined C≡C and C−C bonds) is higher than that in graphene (280 ~ 300 THz, due to the *sp*^2^-hybridized carbon bonds^38^). This partially contributes to the higher *κ* value of carbyne (Fig. 2), since the contribution to *κ* from optical phonon is significant in nanostructures^39^. Moreover, in graphene, the out-of-plane optical (ZO) phonon mode (Γ to M direction) crosses the in-plane longitudinal acoustic (LA) mode, which could lead to strong phonon-phonon scattering between those two modes. The phonon branches in carbyne are clearly separated from each other without overlapping (except the contact at the boundary of the first Brillouin zone), which could greatly reduce phonon-phonon scattering and also explains the ultrahigh *κ* value for carbyne (Fig. 2). Finally, the total number of excitable phonon modes (number of k-points, *N*_k−point_ = *N*_atom_, times 6 branches) in carbyne is limited due to the simple 1D nature. In comparison, 2D graphene^40^ and various 1D polymers^41^ exhibit highly overlapped and complicated phonon dispersion patterns with a lot more excitable modes.

The phonon dispersion for cumulene (Fig. 1(b)) mimics the simplest possible monoatomic chain. LD calculations also miss the TA modes and slightly over-predict the LA mode frequency compared to MD simulation results. The dominant (and also the highest frequency) peak (360 THz from MD) in the longitudinal phonon DOS corresponds to the effective spring constant of C = C (*C*_2_) (see Fig. 1(b) and Supplemental Information). This high frequency LA branch in cumulene partially contributes to its higher *κ* value than graphene, in addition to its simplest possible phonon dispersion.

**Figure 3:**
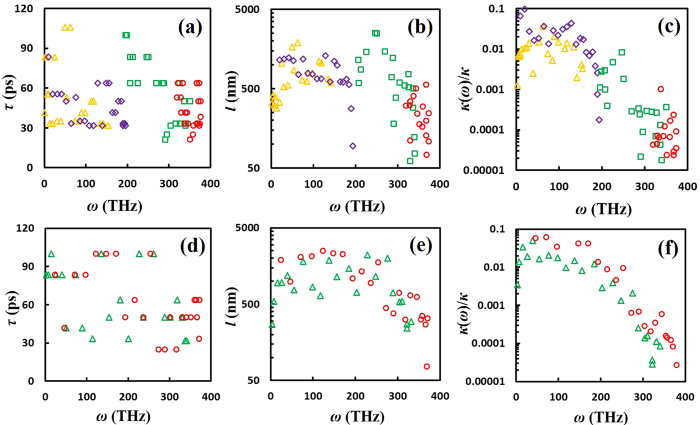
Predicted spectral phonon lifetime, MFP, and thermal conductivity for carbyne and cumulene. (**a**,**d)** phonon lifetime *τ*_*p*,*k*_, **(b**,**e)** MFP *l*_*p,k*_, **(c**,**f)** spectral contribution *κ*(*ω*) to *κ* as a function of *ω* for carbyne (top row) and cumulene (bottom row). The same color and symbol codes in Fig. 1 are applied here.

The predicted spectral phonon lifetimes *τ*_*p*,*k*_ with respect to phonon angular frequency *ω* are shown in Fig. 3(a–d) for carbyne and cumulene. For all phonon branches, the *τ*_*p*,*k*_ values stay within the range of 30 ~ 110 ps and are much longer than those for monolayer graphene (about 0 ~ 30 ps at 300 K)^13^, owing to the reduced phonon-phonon scattering from non-overlapped dispersion curves. The longer *τ*_*p*,*k*_ for both carbon chains can partially contribute to their higher *κ* value compared to monolayer graphene. Interestingly, *τ*_*p*,*k*_ for both carbon chains also exhibit relatively weak dependence on frequency, similar to that for monolayer graphene^13^. Such trend in *τ*_*p*,*k*_ differs from the traditional case in which it exhibits a quick decline as *ω* increases and follows a scaling law of *τ*_*p*,*k*_ ~ *ω*^−*α*^ based on the three-phonon scattering model^42^,^43^. This weak dependence observed here can be attributed to anharmonic events at room temperature (not considered in the above scaling law) or the one-dimensional nature similar to carbon nanotubes^44^. Comparing carbyne and cumulene, the average value of *τ*_*p*,*k*_ for carbyne (~50 ps) is slightly shorter than that for cumulene (~60 ps).

Figure 3(b–e) illustrate calculated spectral phonon MFP *l*_*p,k*_ as a function of frequency *ω* in log scales. For carbyne, *l*_*p,k*_ ranges primarily from 500 ~ 2000 nm for LA and TA branches, while it is below 500 nm for the LO branch. Phonon MFPs of all acoustic modes are longer than that of the LO mode due to their higher group velocities (see Fig. 1(a)). *l*_*p,k*_ for TO branch stays in a much wider range of 50 ~ 2500 nm. For both branches (LA and TA) in cumulene, the *l*_*p,k*_ values are within the similar range of 500 ~ 2500 nm for *ω* below 250 THz, and decreases when *ω* is above 250 THz. It is worth noting that *l*_*p,k*_ of the LA and TA branches for both carbon chains are much larger than those for monolayer graphene that ranges from only 10 ~ 600 nm^13^.

Using the kinetic theory of phonon transport (Eq. (2)), we can obtain the spectral contribution, *κ*(*ω*), from each phonon mode to the total thermal conductivity *κ*. As shown in Fig. 3(c,f), the normalized contribution *κ*(*ω*)/*κ* is reduced when increasing *ω*, and the contributions from low- and medium-frequency (<200 THz) acoustic modes dominate the total thermal conductivity. High-frequency optical modes also contribute to *κ*, although such contribution is limited. Among acoustic modes, spectral contributions to *κ* from the LA mode are larger than that from the TA mode for both carbon chains, and therefore, are the primary contributors to *κ* in both carbon chains. Since *l*_*p,k*_ in the frequency domain of <200 THz are within the micron-range, we can conclude that those acoustic modes can transport ballistically along micron-length carbyne or cumulene chains.

### Tensile Deformation Effects on Phonon Transport

**Figure 4:**
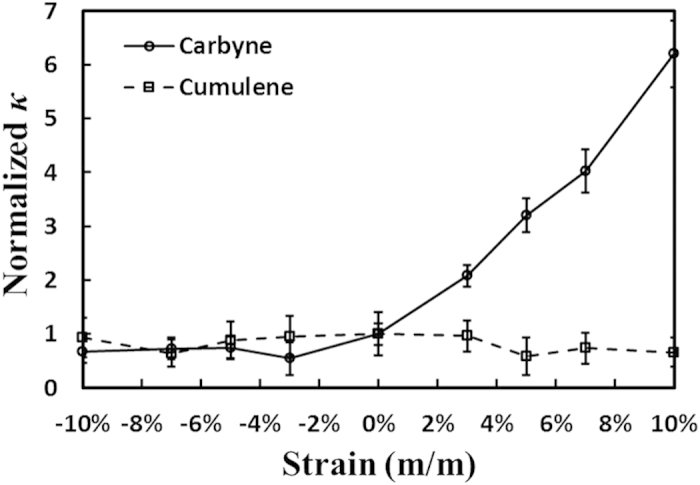
Tensile deformation effects on thermal transport in carbyne and cumulene. Normalized *κ* as a function of tensile strain for carbyne and cumulene. Standard errors in *κ* value are in the range of 20% ~ 60% based on three independent simulations.

The effects of tensile deformations (extension or compression along the chain axis) on *κ* for both carbon chains are shown in Fig. 4. Engineering strains from *ε* = −10 to 10% are applied to both chains under a constant *T*_MD_ = 300 K, and the resulting *κ* are normalized against the originally un-deformed 0% strain case. Extensions of carbyne significantly enhance *κ* and lead to a positive, close-to-linear correlation with strain, in contrast to previous reports of reduced *κ* values in nanostructures under tensile extensions^45^,^46^. The phonon dispersion and DOS for carbyne under 10% strain (Supplementary Fig. S3(a)) exhibit LA mode softening (red shift in frequencies) and group velocity reduction compared to the unstrained case, in consistent with previous reports^45^,^46^. However, the peak heights in the LA phonon DOS increase in the range of 140 ~ 180 THz. TA and TO phonon modes under 10% strain remain similar to the unstrained case with small fluctuations in dispersion curves and group velocities (Supplementary Fig. S3(a)). LO phonons in carbyne under 10% strain do not exhibit any mode softening, but the DOS becomes broader and their peaks heights increase in the range of 320 ~ 370 THz. Interestingly, under the 10% strain, a small phonon bandgap of 15 THz is opened between the LA and the TO modes at the first Brillouin zone (Supplementary Fig. S3(a)), compared to the unstrained case in which the two modes contact at the first Brillouin zone (see Fig. 1(a)).

**Figure 5:**
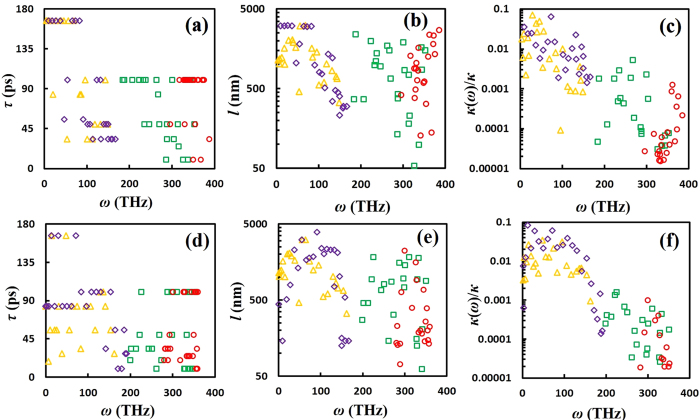
Predicted spectral phonon lifetime, MFP, and thermal conductivity for carbyne under tensile deformations. **(a)** Phonon lifetime *τ*_*p*,*k*_, **(b)** mean free path *l*_*p,k*_, and **(c)** spectral contribution *κ*(*ω*) to *κ* as a function of *ω* for carbyne under 10% extension (top row). **(d)** *τ*_*p,k*_, **(b)** *l*_*p,k*_, and **(c)** *κ*(*ω*)/*κ* as a function of *ω* for carbyne under 10% compression (bottom row). The same color and symbol codes in Fig. 1(a) are applied here.

As shown in Fig. 5(a), the averaged *τ*_*p*,*k*_ for carbyne under 10% strain is about 90 ps, much higher than that in the unstrained case (~60 ps). This can be attributed to the fact that larger phonon bandgap between non-overlapped LA and TO modes (Supplementary Fig. S3(a)) could lead to longer phonon lifetime and reduced phonon-phonon scattering. Therefore, the increases in phonon lifetime and DOS peak heights in the LA mode lead to the enhanced thermal conductivity of carbyne under tensile stretching. The enhanced averaged *τ*_*p*,*k*_ (~70 ps) is also observed in carbyne under 10% compression (see Fig. 5(d). However, significant phonon mode softening in the two acoustic modes (Supplementary Fig. S3(b) for −10% strain) acts against the enhanced *τ*_*p*,*k*_, and therefore, finally leads to similar *κ* values as the unstrained case. The resulting averaged *l*_*p,k*_ (see Fig. 5(b–e)) increase under both tensile stretching and compression cases with an increased upper bound of 3500 nm. Interestingly, under the 10% strain *κ*(*ω*)/*κ* from the TA mode is significantly enhanced in the low-frequency domain (<100 THz), and even outweighs that from the LA mode (see Fig. 5(c)). This makes the TA mode contribution weigh as much as the LA mode contribution over the full phonon spectrum. Such behavior is not observed in carbyne under the −10% strain (see Fig. 5(f)).

**Figure 6:**
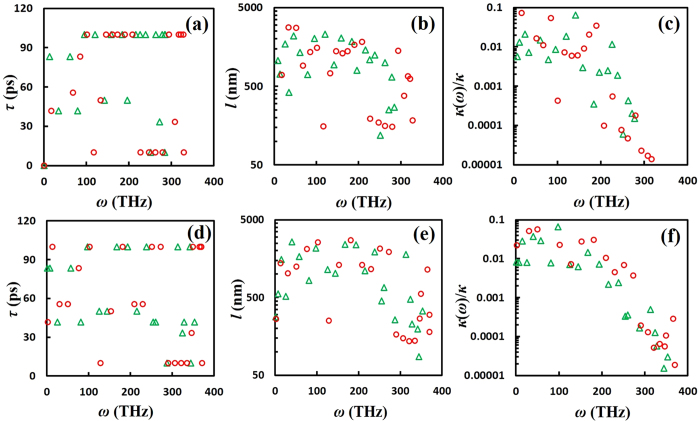
Predicted spectral phonon lifetime, MFP, and thermal conductivity for cumulene under tensile deformations. **(a)** Phonon lifetime *τ*_*p*,*k*_, **(b)** mean free path *l*_*p,k*_, and **(c)** spectral contribution *κ*(*ω*) to *κ* as a function of *ω* for cumulene under 10% extension (top row). **(d)** *τ*_*p*,*k*_, **(b)** *l*_*p,k*_, and **(c)** *κ*(*ω*)/*κ* as a function of *ω* for cumulene under 10% compression (bottom row). The same color and symbol codes in Fig. 1(b) are applied here.

For cumulene, the averaged *τ*_*p*,*k*_ increases to about 90 ps under 10% extension (Fig. 6(a)), and about 65 ps under 10% compression (Fig. 6(d)). However, both deformation cases exhibit significant reductions in group velocity in the low frequency range <180 THz (Supplementary Fig. S4), resulting in very weak dependence of *κ* with respect to strain values. The predicted *l*_*p,k*_ and *κ*(*ω*)/*κ* under the 10% strain exhibit red shifts (see Fig. 6(b,c)), in consistent with the observed phonon mode softening in both the LA and TA modes under 10% strain (see Supplementary Fig. S4(a)). Combining the above competing effects on *τ*_*p*,*k*_, phonon DOS, and group velocity, the averaged *l*_*p,k*_ and *κ*(*ω*)/*κ* for both deformation cases remain similar to that of the unstrained case (see Fig. 6(b,c,e,f)).

## Conclusions

In conclusion, using equilibrium MD simulations and the Green-Kubo approach, we predict the ultrahigh thermal conductivities of carbyne and cumulene. Such conductivity is attributed to the high phonon energies and group velocities in both materials, as well as the reduced phonon-phonon scattering due to the simplest possible phonon dispersion patterns and the resulting non-overlapped phonon branches. Acoustic phonon modes dominate the thermal transport in both carbyne and cumulene, among which the LA mode is the primary contributor to the total thermal conductivity. Prolonged 10 ~ 110 ps spectral phonon lifetime and micron-range (0.5 ~ 2.5 μm) spectral phonon MFP were observed for carbyne and cumulene, which allows ballistic thermal transport along micron-length carbon chains. Interestingly, tensile extensions can enhance the thermal conductivity of carbyne, partially due to the enhanced phonon DOS intensity in the low-frequency acoustic modes. In addition, the enhanced averaged phonon lifetime, as a result of the small phonon bandgap opening at the first Brillouin zone, further increase the thermal conductivity. This finding could serve as an important theoretical guideline for engineering and tuning phonon band structures (e.g., bandgap opening) through materials tensile deformations. We believe that this report will inspire experimental measurements and validations of the predicted ultrahigh thermal conductivities in 1D atomic chains, including carbyne, cumulene, and single BN chains (not reported here but is also expected to have an ultrahigh thermal conductivity), as well as practical deployments of 1D atomic chains in nano-electronic/spintronic devices. In particular, with the very rapid progress of synthesizing long, encapsulated 1D atomic chains in carbon nanotubes (1D chain@CNT)^20^,^21^,^47^,^48^,^49^,^50^, these new hybrid materials could lead to very stable and highly efficient thermal dissipation junctions, metal-metal/metal-semiconducting junctions, and diodes in nano-electronics.

## Methods

### Molecular dynamics simulations

MD simulations and phonon property calculations are carried out using the LAMMPS software package^51^. A second generation force field, PCFF^52^,^53^, is used to model single carbyne and cumulene chains due to its capability of capturing the anharmonic bonding between two carbon atoms, as well as the bending stiffness of the chain through three-body angular potentials to avoid diverging displacements in FPU chains^28^. The force field parameters are listed in Table S1 with a pairwise interaction cutoff distance of 1 nm. The *x* and *y* dimensions are fixed at 2 nm. Different simulation cell size along the *z*-axis lead to carbon chains of 3.3 to 26.2 nm long. After energy minimization (conjugate-gradient) with stress relaxation along the chain direction, *NVT* ensemble using the Nose-Hoover thermostat^54^,^55^ under different temperatures is applied to the single chain system. Enough spacing (~2 nm) is left around the single chain to avoid chain-chain interactions through the periodic image. A time step of 1 fs is used for all the MD simulations here.

### Thermal conductivity calculations

The Green-Kubo approach^6^ is used to predict thermal conductivities along the chain axis. After energy minimization, NVT simulations at different temperatures (1 to 1000 K) were conducted for 30 ns each, followed by 50 ns to integrate the heat flux autocorrelation function (HFACF) for computing *κ*:


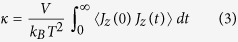


where *k*_B_ is the Boltzmann constant, *V* is the system volume (the carbon chain length *L* times its cross-sectional area of 3.35 × 3.35 Å^2^ 56), and *J*_*z*_ is the heat flux along the *z*-axis.

### Intrinsic spectral phonon property calculations

The velocity autocorrelation function (VAF) averaged over all atoms is collected every 5 fs from a 10 ps NVT simulation to obtain the normalized phonon DOS as a function of angular frequency *ω*, *D*_*j*_(*ω*), along the direction *j* = *x*, *y* or *z*, by Fourier transforming the VAF^57^,^58^,^59^. Specifically,


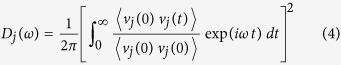


where *v*_*j*_ is the atomistic velocity along the direction *j*.

Phonon dispersion relations are computed based on the dynamical matrix obtained from the fluctuation-dissipation theory^60^ using data collected from 5 ns of NVT simulation. Although we focus on phonon transport in 1D along the chain axis, phonon DOS and dispersion are computed in 3D and decomposed into contributions from *z* (L, longitudinal) and *x*/*y* (T, transverse) dimensions. The absolute group velocity |*v*_*p,k*_|, as a function of *p* (polarization) and *k* (wave vector), can be computed from the gradients of the phonon dispersion curves in Fig. 1: |*v*_*p,k*_| = |∂*ω*_*p,k*_/∂*k*|. For comparison, analytical LD calculations are also carried out in 1D to predict phonon dispersion, DOS (*D*_*p,k*_ = (*π*∂*ω*_*p,k*_/∂*k*)^−1^), and |*v*_*p,k*_| (see Supplementary Information).

Spectral phonon lifetime, *τ*_*p,k*_, is calculated from spectra energy density (SED) *Φ*_*p,k*_ (*ω*) using the expression^61^:


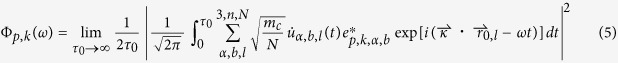


where *n* is number of atoms in the unit cell (*n*_*carbyne*_ = 2 and *n*_*cumulene*_ = 1); *N* is the total number of atoms; *m*_*c*_ is the mass of carbon atom; 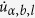 is the *α*-component of the velocity of the *b*^*th*^ atom in the *l*^*th*^ unit cell; 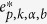 is the complex conjugate of the eigenvector associated with *b*^*th*^ atom for the phonon mode (*p*,*k*); 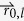 is equilibrium position vector of the *l*^*th*^ unit cell; 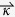 (= *k* for the 1D carbon chain considered here) is the wave vector; *τ*_*0*_ is the total simulation time. SED satisfies a Lorenzian distribution^62^, namely,


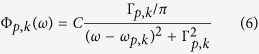


where *ω*_*p,k*_ is the eigen-frequency for the phonon mode (*p*,*k*); Γ_*p,k*_ corresponds to the half width at half maximum of SED. After fitting SED with a Lorenzian function, the phonon lifetime *τ*_*p*,*k*_ can be calculated by *τ*_*p*,*k*_ = 1/2Γ_*p,k*_. Spectral phonon MFP *l*_*p,k*_ can be computed based on its definition: *l*_*p,k*_ = *v*_*p,k*_·*τ*_*p*,*k*_.

## Additional Information

**How to cite this article**: Wang, M. and Lin, S. Ballistic Thermal Transport in Carbyne and Cumulene with Micron-Scale Spectral Acoustic Phonon Mean Free Path. *Sci. Rep.* **5**, 18122; doi: 10.1038/srep18122 (2015).

## Supplementary Material

Supplementary Information
