# Supplementary material for: Ballistic Thermal Transport in Carbyne and Cumulene with Micron-Scale Spectral Acoustic Phonon Mean Free Path
Source: Sci Rep. 2015 Dec 10;5:18122. doi: 10.1038/srep18122 (PMC4674807; doi:10.1038/srep18122)
Supplement: Supplementary Information [file srep18122-s1.pdf]

Supplementary Information

Ballistic Thermal Transport in Carbyne and Cumulene with Micron-Scale Spectral Acoustic Phonon Mean Free Path

Mingchao Wang and Shangchao Lin*

Department of Mechanical Engineering, Materials Science and Engineering Program, FAMU-FSU College of Engineering, Florida State University, Tallahassee, Florida 32310, USA

*Corresponding author contact information: slin@eng.fsu.edu

Table S1. Force field parameters for covalent bonding interactions in carbyne and cumulene. The 1-4 non-bonded interactions between carbon atoms were modeled by the 12-6 Lennard-Jones potential with $\sigma = 4.01$ Å and $\varepsilon = 0.064$ kcal/mol. The C-C bond and C=C bond were modeled using a 4th-order polynomial function (anharmonic), while the C≡C bond was modeled using the common quadratic function (harmonic) since it is very stiff. The equation for the anharmonic covalent bonding energy is: $E(r) = K_2(r - r_0)^2 + K_3(r - r_0)^3 + K_4(r - r_0)^4$. Experimentally interpolated bond lengths are shown in the parentheses[1]. An equilibrium angle of 180° and harmonic spring constant of 200 kcal/mol was used for angular bending in both carbyne and cumulene.

Bond Types	r_0 (Å)	K_2 (kcal/mol/Å^2)	K_3 (kcal/mol/Å^3)	K_4 (kcal/mol/Å^4)
C–C in carbyne	1.530 (1.380)	299.67	–501.77	679.81
C≡C in carbyne	1.204 (1.207)	800.00	–	–
C=C in cumulene	1.340 (1.282)	543.99	–1238.20	1644.03

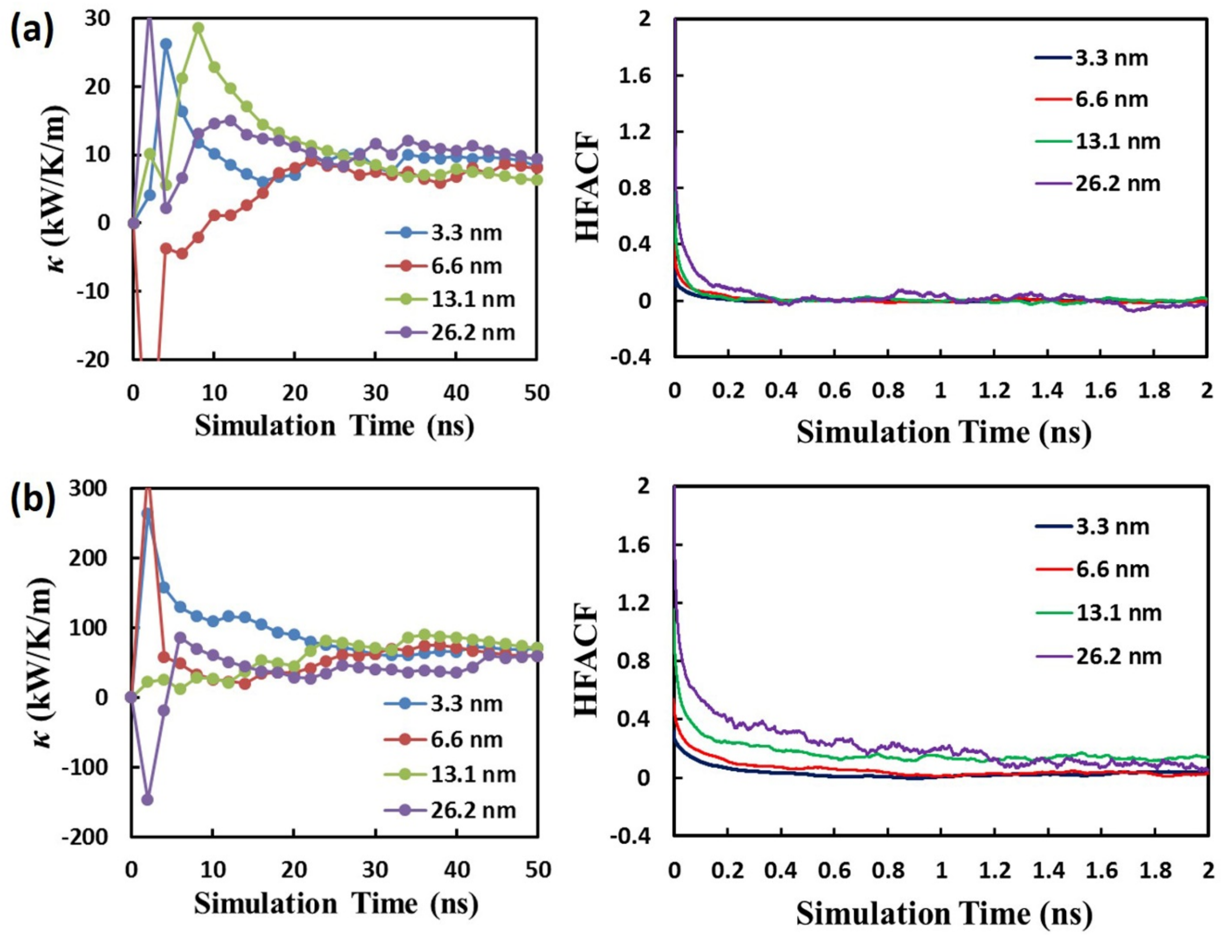

FIG S1. Time evolutions of Green-Kubo MD simulation predicted thermal conductivity *κ* (left column, running average) and HFACF (right column, for the last 2 ns) for **(a)** carbyne and **(b)** cumulene chains of various lengths, all at T_{MD} = 300 K.

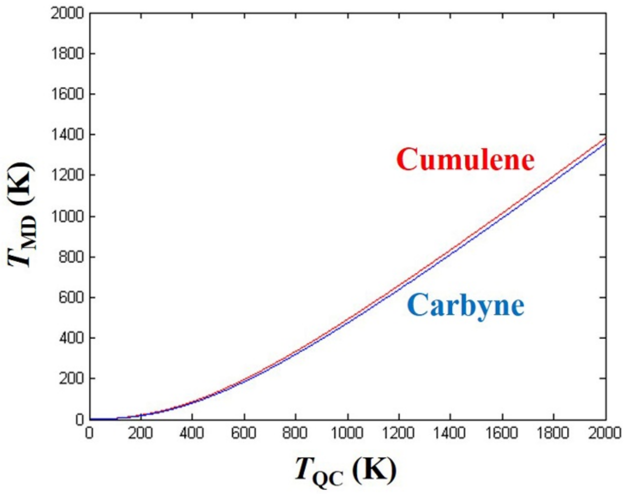

FIG S2. Conversion curves for carbyne and cumulene from MD (T_{MD}) to quantum-corrected (T_{QC}) temperatures.

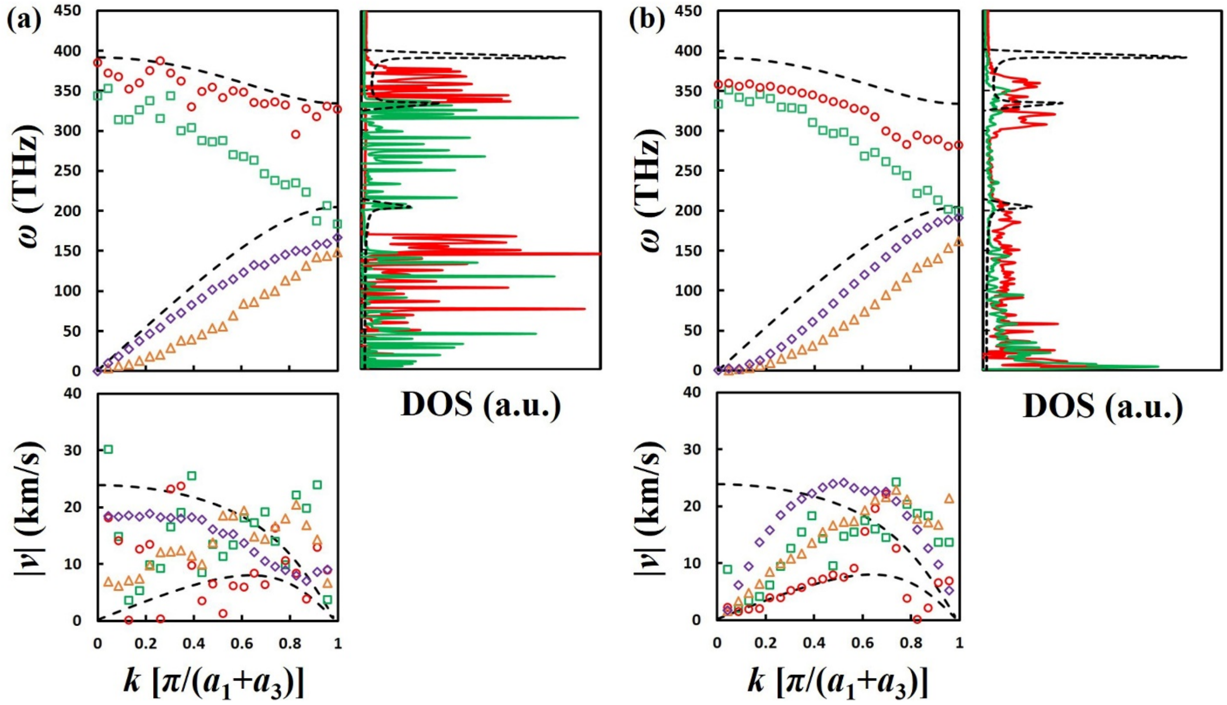

FIG S3. Phonon dispersion (top left), DOS (top right) and group velocity (bottom) of the carbyne chain under **(a)** 10% extension and **(b)** 10% compression. The computational method and color code are the same as in **Fig. 1 (a)**.

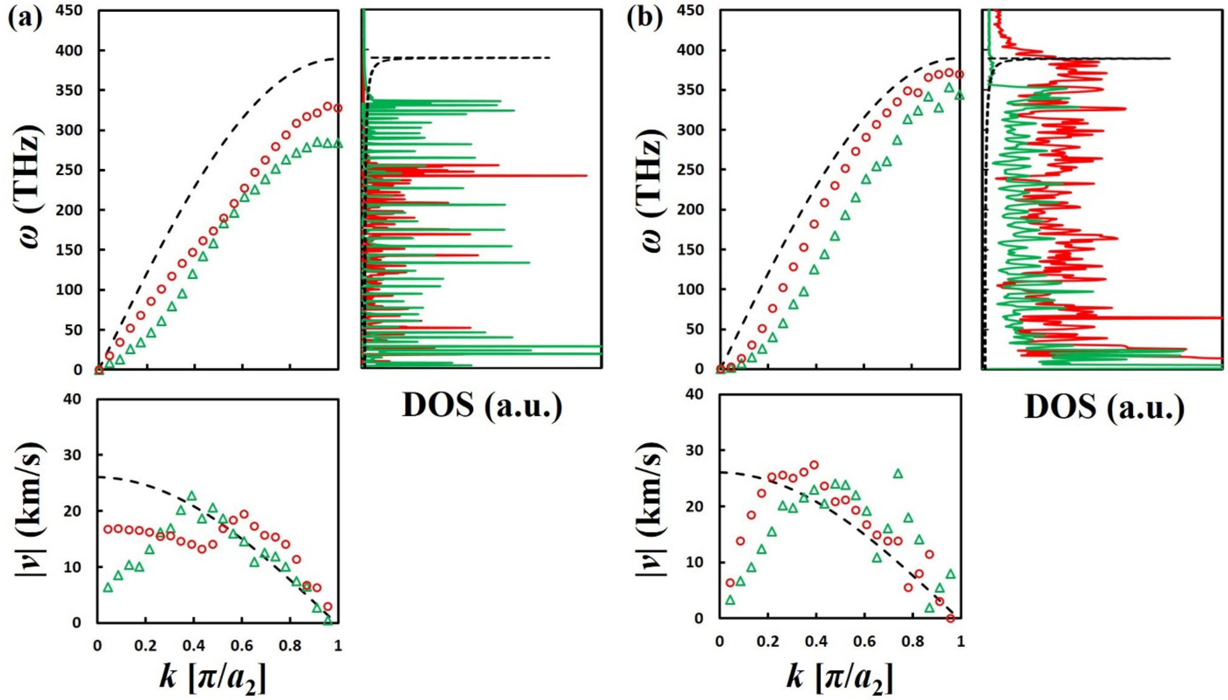

FIG S4. Phonon dispersion (top left), DOS (top right) and group velocity (bottom) of the cumulene chain under **(a)** 10% extension and **(b)** 10% compression. The computational method and color code are the same as in **Fig. 1 (b)**.

Analytical Lattice Dynamics (LD) Calculations

The single carbyne chain can be modeled as a monoatomic spring consisting of beads of the same mass m but two alternating spring constants C_1 (for C–C) and C_3 (for C≡C) as well as the corresponding equilibrium separations a_1 and a_3 (**Fig. 1 (a)** in the main text). The classical Newton's second law of motion applied to this chain leads to the following equation for the displacement on particles 2n and 2n+1, u_{2n} and u_{2n+1}, from force balancing:

$$m\frac{\partial^2 u_{2n}}{\partial t^2} = C_3\left(u_{2n+1} - u_{2n}\right) - C_1\left(u_{2n} - u_{2n-1}\right) \tag{S1}$$

$$m\frac{\partial^2 u_{2n+1}}{\partial t^2} = C_1\left(u_{2n+2} - u_{2n+1}\right) - C_3\left(u_{2n+1} - u_{2n}\right) \tag{S2}$$

We propose the following complex wave equations as the general solutions to u_{2n} and u_{2n+1}:

$$u_{2n} = U_1 \exp\{i[nk(a_1 + a_3) - \omega t]\} \tag{S3}$$

$$u_{2n+1} = U_2 \exp\{i[nk(a_1 + a_3) + ka_3 - \omega t]\} \tag{S4}$$

Substituting Eqs. S3 and S4 into Eqs. S1 and S2 gives:

$$-m\omega^2 U_1 = C_3[U_2 \exp(ika_3) - U_1] - C_1[U_1 - U_2 \exp(-ika_1)] \tag{S5}$$

$$-m\omega^2 U_2 = C_1[U_1 \exp(ika_1) - U_2] - C_3[U_2 - U_1 \exp(-ika_3)] \tag{S6}$$

which can be rearranged into:

$$\left[m\omega^2 - (C_1 + C_3)\right]U_1 + \left[C_1 \exp(-ika_1) + C_3 \exp(ika_3)\right]U_2 = 0 \tag{S7}$$

$$\left[m\omega^2 - (C_1 + C_3)\right]U_2 + \left[C_1 \exp(ika_1) + C_3 \exp(-ika_3)\right]U_1 = 0 \tag{S8}$$

By canceling out U_1 or U_2, we got:

$$m^2\omega^4 - 2m(C_1 + C_3)\omega^2 + C_1^2 + C_3^2 + 2C_1C_3 = C_1^2 + C_3^2 + 2C_1C_3 \cos[k(a_1 + a_3)] \tag{S9}$$

which can be further simplified to:

$$m^2\omega^4 - 2m(C_1 + C_3)\omega^2 + 2C_1C_3\{1 - \cos[k(a_1 + a_3)]\} = 0 \tag{S10}$$

which leads to the phonon dispersion relation:

$$\omega = \sqrt{\frac{(C_1 + C_3) \pm \sqrt{C_1^2 + C_3^2 + 2C_1C_3 \cos[k(a_1 + a_3)]}}{m}} \tag{S11}$$

From the PCFF force field used for carbyne, the anharmonic C–C bond possesses a leading order harmonic spring constant of C_1 = 299.67×2 kcal/mol/Å^2, while the C≡C bond is quite harmonic with a spring constant of C_3 = 800.00×2 kcal/mol/Å^2. Other parameters can be obtained from the force field: a_1 = 1.530 Å, a_3 = 1.204 Å and m = 12 g/mol. At the long wavelength limit (k = 0), the acoustic phonon dispersion is $\omega_{optical}(k = 0) = 0$ and the optical phonon dispersion becomes:

$$\omega_{optical}(k=0)=\sqrt{\frac{2(C_1+C_3)}{m}}=\omega_{C_1+C_3} \tag{S12}$$

where (C_1 + C_3) is the effective spring constant. Similarly, at the first Brillouin zone ($k = \pi/(a_1+a_3)$), the acoustic and optical phonon dispersion become:

$$\omega_{acoustic}\left(k=\frac{\pi}{a_1+a_3}\right)=2\sqrt{\frac{C_1}{2m}}=\omega_{C_1} \tag{S13}$$

$$\omega_{optical}\left(k=\frac{\pi}{a_1+a_3}\right)=2\sqrt{\frac{C_3}{2m}}=\omega_{C_3} \tag{S14}$$

Similarly, a single cumulene chain can be approximated as a monoatomic spring of mass m, spring constant C_2 (for C=C), and equilibrium separation a_2 (**Fig. 1 (b)** in the main text). The analytical solution for phonon dispersion of this chain is well-known as:

$$\omega=2\sqrt{\frac{C_2}{m}}\left|\sin\left(\frac{ka_2}{2}\right)\right|=\omega_{C_2} \tag{S15}$$

From the PCFF force field used for cumulene, the anharmonic C=C bond possesses a leading order harmonic spring constant of C_2 = 543.99×2 kcal/mol/Å^2, equilibrium separation of a_2 = 1.340 Å, and mass of m = 12 g/mol.

Supplemental References

1. Kastner, J. *et al.* Reductive preparation of carbyne with high yield. An in situ raman scattering study. *Macromolecules* **28**, 344-353 (1995).
